# Geographic Access to Diabetes Prevention Program Sites: New York State Department of Health

**DOI:** 10.5888/pcd11.130400

**Published:** 2014-02-27

**Authors:** Rachael A. Ruberto, Ian F. Brissette

**Affiliations:** **Author Affiliation:** Ian F. Brissette, New York State Department of Health, Albany, New York

## Background

New York’s Division of Chronic Disease Prevention has supported the delivery of the Diabetes Prevention Program (DPP) through community-based organizations across the state. The DPP is a 16-week lifestyle change program designed to prevent or delay type 2 diabetes in adults who are at high risk for the disease. In 2009, 10 YMCA sites across the state were selected to deliver the program. In subsequent years, additional community-based organizations developed capacity to deliver DPP. The goal of the New York State DPP is to establish critical partnerships that serve as a statewide infrastructure of effective, accessible, and affordable diabetes prevention programs.

## Methods

New York’s Bureau of Chronic Disease Evaluation and Research used a geographic information system (GIS) to identify counties with high prediabetes risk-factor composite scores based on county-level prevalence of obesity, physical inactivity, hypertension, and diagnosed diabetes (Figure). The standardized z-score was calculated by using data from the 2008–09 New York State Behavioral Risk Factor Surveillance System (1) and the 2009 New York City Community Health Survey (2), and ranged from −6.01 (lowest risk) to 7.06 (highest risk) among New York State (NYS) counties. Spatial, street-level network analysis was used to calculate 30-minute drive-time buffers around existing DPP sites. Tract-level data from the 2010 US Census (http://www.census.gov/2010census/) was used to calculate the percentage of the NYS population living within the buffers. Potential DPP sites were identified by using 2011 data indicating the locations of federally qualified health centers, independent living centers, and area agencies on aging.

**Figure:**
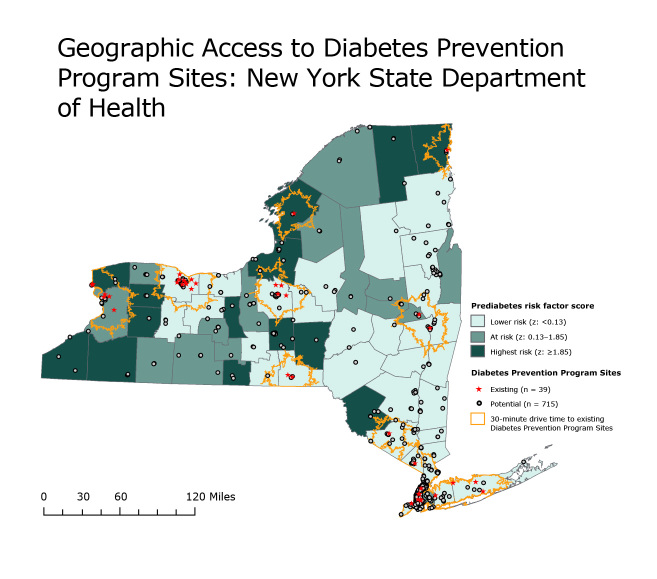
Interactive map of existing and potential Diabetes Prevention Program sites in New York State, by county. Data sources: Source for prediabetes risk factor score: New York State Behavioral Risk Factor Surveillance System (1) and New York City Community Health Survey (2). Source for existing diabetes prevention program sites: New York City Department of Health and Mental Hygiene (3), New York State Department of Health (4), and Centers for Disease Control and Prevention (5). Source for potential program sites: Health Resources and Services Administration (6), New York Association for Independent Living (7,8), and New York State Office for the Aging (9).

## Main Findings

Although approximately 80.0% of New York’s population resides within a 30-minute drive time to a Diabetes Prevention Program site; most people living in 10 of the counties with the highest prediabetes risk-factor score live at a distance beyond a 30-minute drive to an existing site.

## Action

The expansion of DPP sites through additional community-based facilities would improve access to prevention programs in communities with high diabetes risk-factor profiles and would facilitate the improvement of community–clinical linkages across the state to sustain prevention efforts for type 2 diabetes. The New York State Department of Health has used information from GIS analysis to identify areas with limited access to DPPs and encourage key partners to locate programs in community-based organizations serving at-risk populations. Future plans include expanding the analysis to incorporate all evidence-based chronic disease programs and assessing demographic differences among populations living within and outside the 30-minute drive-time radius to existing program sites.
